# The impact of insurance status on psoriasis patients’ healthcare-seeking behavior: a population-based study in the United States

**DOI:** 10.1186/s12913-024-11992-z

**Published:** 2024-11-29

**Authors:** Kaviyon Sadrolashrafi, Audrey Hao, Rebecca K. Yamamoto, Lily Guo, Robin Kikuchi, Hannah C. Tolson, Sara N. Bilimoria, Danielle K. Yee, April W. Armstrong

**Affiliations:** 1 Kirk Kerkorian School of Medicine at UNLV, Las Vegas, USA; 2 Keck School of Medicine of University of Southern California, Los Angeles, USA; 3 Georgetown University School of Medicine, Washington, DC USA; 4 Duke University School of Medicine, Durham, USA; 5 The University of Arizona College of Medicine – Phoenix, Phoenix, USA; 6 Division of Dermatology, Department of Medicine, David Geffen School of Medicine at UCLA, 200 Medical Plaza, Los Angeles, CA 90095 USA

**Keywords:** Psoriasis, Access to care, Delayed care, Health insurance, Health equity, Healthcare-seeking behavior, Medical Expenditure Panel Survey, MEPS, Medicare, Medicaid

## Abstract

**Background:**

Psoriasis is a chronic, inflammatory skin condition requiring long-term care. However, many psoriasis patients may not regularly receive care. Several factors affect access to care in the United States, including health insurance status. Additionally, it is unknown how health insurance status impacts the healthcare-seeking behavior of psoriasis patients. Healthcare-seeking behavior is broadly defined as an individual’s actions to prevent or treat a perceived health problem, such as visiting a physician’s office. Because early diagnosis and timely treatment improve patient outcomes, determining how insurance status impacts psoriasis patients’ healthcare-seeking behavior and their ability to get care is important. This allows us to identify patients at risk for being untreated or undertreated. In this study, we aimed to assess the relationship between insurance status and (1) the degree to which psoriasis patients delay seeking or receiving care and (2) the degree to which psoriasis patients are unable to obtain care.

**Methods:**

This population-based study used 20 years of data from the Medical Expenditure Panel Survey from 2002 to 2021. We calculated descriptive statistics and performed adjusted multivariable logistic regression analyses.

**Results:**

We identified a weighted total of 4,506,850 psoriasis patients. Compared to those with private insurance, psoriasis patients with public-only insurance were 2.7 times more likely to delay seeking or receiving care (95% CI, 1.26–5.87). Compared to private insurance patients, uninsured psoriasis patients were 3.4 times more likely to be unable to obtain care (95% CI, 1.31–8.92). Compared to those with public-only insurance, uninsured psoriasis patients were 3.7 times more likely to be unable to obtain care (95% CI, 1.32–10.38).

**Conclusions:**

This study found that psoriasis patients with public-only insurance were significantly more likely to delay seeking or receiving care compared to those with private insurance. This study also found that uninsured psoriasis patients were significantly more likely to be unable to obtain care than psoriasis patients with private insurance and those with public-only insurance. Developing strategies to increase healthcare access is necessary to ensure equitable, timely, and appropriate care for all psoriasis patients, regardless of their insurance status.

## Background

 Psoriasis is a chronic, inflammatory skin condition that requires long-term management [1–5]. Early diagnosis and timely treatment may mitigate symptoms, improve quality of life, and prevent the development of comorbidities associated with psoriasis [6–10]. However, some psoriasis patients do not regularly receive care. Nontreatment and undertreatment are common trends among United States psoriasis patients, which may contribute to increased disease morbidity and decreased quality of life [5, 11–13].

Healthcare-seeking behavior is broadly defined as the actions an individual takes to prevent or treat a perceived health problem [14–17]. Healthcare-seeking behavior encompasses the decision to seek care and the timing of services used [14–17]. Many factors influence healthcare-seeking behavior, such as healthcare accessibility [14–17]. Several factors affect access to care in the United States, including health insurance status [18].

Differences in access to dermatological care based on insurance type are described in the literature [19–21]. Some dermatology clinics accept fewer new patients because of their insurance type, which may prolong appointment wait times and delay treatments [19–21]. Accessible care is essential for managing chronic conditions like psoriasis, which necessitates routine screening and dermatologist follow-up [6]. It is important to be aware of factors affecting psoriasis patients’ decision to seek care and their ability to receive care. An enhanced understanding of these factors will enable dermatologists to identify populations at risk of being untreated or undertreated. This information can help form policies that increase access to care, guide outreach initiatives that promote health-protective behaviors, and ultimately improve patient outcomes [15, 16].

There is a paucity of literature evaluating the impact of health insurance status on psoriasis patients’ healthcare-seeking behavior and their ability to receive care. Our study aims to examine this influence by assessing the relationship between psoriasis patients’ health insurance status and (1) the degree to which psoriasis patients delay seeking or receiving care and (2) the degree to which psoriasis patients are able to obtain care.

## Methods

### Study population

This cross-sectional, population-based study used data from the Medical Expenditure Panel Survey (MEPS) from 2002 to 2021. The Agency for Healthcare Research and Quality (AHRQ) constructed this nationally representative database to provide estimates of the civilian, noninstitutionalized United States population [22]. We used the Full-Year Consolidated and Medical Conditions Files from the Household Component (HC) of MEPS to identify our study population and obtain data regarding sociodemographic and socioeconomic characteristics, access to care, use of medical services, health conditions, health status, and prescribed medications. This data was collected from a sample of households participating in the previous year’s National Health Interview Survey (NHIS), administered by the National Center for Health Statistics [22, 23].

To define the study population, we used the *International Classification of Diseases*,* Ninth Revision*,* Clinical Modification* (*ICD-9-CM*) code in the Medical Conditions file from 2002 to 2015 and the *International Classification of Diseases*,* Tenth Revision*,* Clinical Modification* (*ICD-10-CM*) code in the Medical Conditions file from 2016 to 2021. Patients with psoriasis were identified using the *ICD-9-CM* code 696 (“Psoriasis/Like Disorders”) and *ICD-10-CM* code L40 (“Psoriasis”); these data represented patient-reported diagnoses of psoriasis. Only patients aged 18 years or older were included in our analysis. Our study population included survey respondents who reported one person in their Reporting Unit (RU). The RU is a data collection unit used in MEPS comprised of related respondents living in the same physical household; therefore, the RU can represent a single person or multiple individuals [22, 24]. This study used publicly available, de-identified data; therefore, it was exempt from Institutional Review Board approval.

### Variables

We assessed (1) the delay in seeking or receiving care and (2) the inability to obtain care using variables from the Access to Care (AC) supplement of MEPS-HC. All variables represented questions with binary responses of “yes” or “no.” Previous studies have used similar questions to assess barriers to healthcare access in the noninstitutionalized United States population [25, 26].

To assess the delay in seeking or receiving care, we combined two MEPS variables into one that we renamed “Delayed in Seeking or Receiving Care” (Table 1). MEPS used MDDLAY42 from 2002 to 2017 and DLAYCA42 from 2018 to 2021.

**Table 1:** Variables in Medical Expenditure Panel Survey used to assess the delay in seeking or receiving care. Our renamed variable, “Delayed in Seeking or Receiving Care,” combines MDDLAY42 and DLAYCA42

MEPS Variable	Question
DLAYCA42^a^	Please think about the last 12 months, that is, between {Month Year-1} and today. {Have you/Has {person}} delayed seeking medical care/Has medical care been delayed for anyone in the household} because of worry about the cost?
MDDLAY42^b^	In the last 12 months, was anyone in the family delayed in getting medical care, tests, or treatments they or a doctor believed necessary?

^a﻿^MEPS variable available from 2018 to 2021

^b﻿^MEPS variable available from 2002 to 2017

To assess the inability to obtain care, we combined two MEPS variables into one that we renamed “Unable to Obtain Care” (Table 2). MEPS used MDUNAB42 from 2002 to 2017 and AFRDCA42 from 2018 to 2021.

**Table 2:** Variables in Medical Expenditure Panel Survey used to assess the inability to obtain care. Our renamed variable, “Unable to Obtain Care,” combines MDUNAB42 and AFRDCA42

MEPS Variable	Question
AFRDCA42^a^	In the last 12 months, was there any time when {{you/{person}}/anyone in the household} needed medical care, but did not get it because {{you/he/she}/they} couldn’t afford it?
MDUNAB42^b^	In the last 12 months, was anyone in the family unable to obtain medical care, tests, or treatments they or a doctor believed necessary?

^a^MEPS variable available from 2018 to 2021

^b^MEPS variable available from 2002 to 2017

We used the single-person RU as an inclusion criterion for our analyses to ensure that responses to MEPS questions reflected psoriasis patients at the individual level. If respondents from a single-person RU answered “yes” to a survey question between 2002 and 2017, their response was coded as “delay in receiving medical care” or “unmet need for medical care,” respectively [27]. In 2018, the language of the survey questions changed slightly. If respondents from a single-person RU completed the survey between 2018 and 2021, questions were displayed as “{{Have you/Has {person}} delayed seeking medical care because of worry about the cost?” or “In the last 12 months, was there any time when {{you/{person}} needed medical care, but did not get it because {{you/he/she}} couldn’t afford it?” [27].

### Data analysis

We calculated descriptive statistics for sociodemographic and clinical characteristics across the weighted cohort. Following MEPS recommendations, individual-level sampling weights were used to best approximate the sampling methods used during survey administration [28]. Continuous variables were reported with mean and standard deviation, and categorical variables were reported with weighted numbers and proportions.

We performed multivariable logistic regression to assess the association between health insurance status and healthcare-seeking behavior, characterized by the binary outcome variables of “Delayed in Seeking or Receiving Care” and “Unable to Obtain Care.” We adjusted all regression models for demographic and clinical characteristics, including age, sex, race, ethnicity, geographical region, and medical comorbidities. We used the Charlson Comorbidity Index (CCI), which estimates mortality risk based on comorbid medical conditions, as a covariate to represent medical comorbidities [29]. Results were reported as an adjusted odds ratio (aOR) with associated confidence intervals (CIs) and *p*-values.

All statistical analyses were performed using Stata 18.0 (StataCorp LP, College Station, TX). The statistical significance threshold for all analyses was set at *p* < 0.05.

## Results

### Cohort characteristics

A weighted total of 4,506,850 psoriasis patients were identified. We stratified psoriasis patients in our weighted cohort into three insurance groups: private insurance (72.5%), public-only insurance (23.6%), and uninsured (3.9%). The sociodemographic and clinical characteristics of psoriasis patients in the total cohort and each insurance group are presented in Table 3.

**Table 3:** Demographic and clinical characteristics of the weighted total cohort of psoriasis patients and psoriasis patients in different insurance groups

Characteristic	Total Cohort (Weighted *N* = 4,506,850)	Private Insurance (Weighted *N* = 3,267,296)	Public-Only Insurance (Weighted *N* = 1,064,306)	Uninsured (Weighted *N* = 175,248)	*p*-value
Age, mean years (SEM)					< 0.001^*^
	54.0 (0.82)	51.5 (0.74)	63.7 (0.54)	40.8 (3.77)	
Sex, *N* (%)					0.025^†^
Male	1,982,948 (44.0)	1,456,561 (44.6)	414,228 (38.9)	112,159 (64.0)	
Female	2,523,902 (56.0)	1,810,735 (55.4)	650,078 (61.1)	63,089 (36.0)	
Race, *N* (%)					0.171^†^
White	3,963,418 (87.9)	2,901,359 (88.8)	906,789 (85.2)	155,270 (88.6)	
Black	312,324 (6.9)	218,909 (6.7)	73,437 (6.9)	19,978 (11.4)	
American Indian/Alaska Native	49,330 (1.1)	16,337 (0.5)	32,993 (3.1)	0 (0)	
Asian/Native Hawaiian/Pacific Islander	119,997 (2.7)	81,682 (2.5)	38,315 (3.6)	0 (0)	
Multiple Races	61,781 (1.4)	49,009 (1.5)	12,772 (1.2)	0 (0)	
Ethnicity, *N* (%)					0.087^†^
Hispanic	338,111 (7.5)	218,909 (6.7)	119,202 (11.2)	0 (0)	
Non-Hispanic	4,168,739 (92.5)	3,048,387 (93.3)	945,104 (88.8)	175,248 (100.0)	
Region, *N* (%)					0.009^†^
Northeast	919,654 (20.4)	617,519 (18.9)	291,620 (27.4)	10,515 (6.0)	
Midwest	858,816 (19.1)	689,399 (21.1)	141,553 (13.3)	27,864 (15.9)	
South	1,747,654 (38.8)	1,267,711 (38.8)	358,671 (33.7)	121,272 (69.2)	
West	980,726 (21.7)	692,667 (21.2)	272,462 (25.6)	15,597 (8.9)	
CCI, mean (SEM)					< 0.001^*^
	1.46 (0.06)	1.29 (0.05)	2.12 (0.06)	0.47 (0.16)	

Estimates are adjusted for survey sampling weights. Statistical significance of *p*-values was set at a threshold of 5%

*CCI *Charlson Comorbidity Index, *SEM *Standard Error of the Mean

^*^Analysis of variance of the differences among psoriasis patients of different ages and CCIs

^†^X^2^ test of the differences among psoriasis patients of different sexes, races, ethnicities, and geographic regions

Overall, 9.7% of the total cohort delayed seeking or receiving care and 4.7% were unable to obtain care. We found that 7.7% of psoriasis patients with private insurance, 14.3% of psoriasis patients with public-only insurance, and 18.9% of uninsured psoriasis patients delayed seeking or receiving care. We found that 4.4% of psoriasis patients with private insurance, 3.6% of psoriasis patients with public-only insurance, and 16.5% of uninsured psoriasis patients were unable to obtain care.

### Association between insurance group and the delay in seeking or receiving care

Table 4 presents multivariable logistic regression analyses comparing the likelihood of delay in seeking or receiving care between psoriasis patients in different insurance groups. We adjusted all models for age, sex, race, ethnicity, geographical region, and CCI.

**Table 4:** Multivariable logistic regression analyses comparing the likelihood of delay in seeking or receiving care between psoriasis patients in different insurance groups, adjusting for demographic covariates and medical comorbidities

Variable	Delayed in Seeking or Receiving Care	
	aOR (95% CI)	*p*-value
Insurance Group		
Private	1 [Reference]	
Public-Only	2.72 (1.26–5.87)	0.012
Uninsured	2.30 (0.68–7.77)	0.175
Insurance Group		
Public-Only	1 [Reference]	
Private	0.37 (0.17–0.79)	0.012
Uninsured	0.85 (0.20–3.63)	0.819
Age	1.01 (0.98–1.04)	0.534
Sex		
Male	1 [Reference]	
Female	1.43 (0.71–2.86)	0.309
Race		
White	1 [Reference]	
Black	0.14 (0.08–0.23)	< 0.001
American Indian/Alaska Native	1.84 (0.54–6.29)	0.323
Asian/Native Hawaiian/Pacific Islander	0.73 (0.36–1.46)	0.362
Multiple Races	2.58 (0.58–11.56)	0.210
Ethnicity		
Hispanic	1 [Reference]	
Non-Hispanic	3.25 (0.72–14.56)	0.122
Region		
Northeast	1 [Reference]	
Midwest	1.97 (0.86–4.53)	0.107
South	1.59 (0.68–3.73)	0.279
West	1.17 (0.44–3.11)	0.754
CCI	0.64 (0.39–1.04)	0.071

Estimates are adjusted for survey sampling weights. Statistical significance of *p*-values was set at a threshold of 5%

*aOR *Adjusted Odds Ratio, *CI *Confidence Interval, *CCI *Charlson Comorbidity Index

Compared to private insurance patients, psoriasis patients with public-only insurance were significantly more likely to delay seeking or receiving care (aOR, 2.72; 95% CI, 1.26–5.87; *p* = 0.012). Although uninsured psoriasis patients were more likely to delay seeking or receiving care than those with private insurance, this association was not statistically significant (aOR, 2.30; 95% CI, 0.68–7.77; *p* = 0.175). There was a numeric difference but no statistically significant difference between public-only insurance and uninsured psoriasis patients in the delay in seeking or receiving care (aOR, 0.85; 95% CI, 0.20–3.63; *p* = 0.819)

### Association between insurance group and the inability to obtain care

Table 5 presents multivariable logistic regression analyses comparing the likelihood of being unable to obtain care between psoriasis patients in different insurance groups. We adjusted all models for age, sex, race, ethnicity, geographical region, and CCI.

**Table 5:** Multivariable logistic regression analyses comparing the likelihood of being unable to obtain care between psoriasis patients in different insurance groups, adjusting for demographic covariates and medical comorbidities

Variable	Unable to Obtain Care	
	aOR (95% CI)	*p*-value
Insurance Group		
Private	1 [Reference]	
Public-Only	0.93 (0.40-2.13)	0.853
Uninsured	3.42 (1.31-8.92)	0.013
Insurance Group		
Public-Only	1 [Reference]	
Private	1.08 (0.47-2.49)	0.853
Uninsured	3.70 (1.32-10.38)	0.014
Age	1.02 (0.98-1.06)	0.350
Sex		
Male	1 [Reference]	
Female	1.05 (0.44-2.52)	0.910
Race		
White	1 [Reference]	
Black	1.17 (0.12-11.59)	0.892
American Indian/Alaska Native	6.06 (1.56-23.62)	0.010
Asian/Native Hawaiian/Pacific Islander	1 (--)	N/A
Multiple Races	2.18 (1.44-3.31)	< 0.001
Ethnicity		
Hispanic	1 [Reference]	
Non-Hispanic	1.62 (0.51-5.10)	0.403
Region		
Northeast	1 [Reference]	
Midwest	7.79 (1.52-39.85)	0.015
South	4.60 (0.97-21.89)	0.055
West	7.41 (1.37-39.99)	0.021
CCI	0.58 (0.37-0.93)	0.025

Estimates are adjusted for survey sampling weights. Statistical significance of *p*-values was set at a threshold of 5%

*aOR *Adjusted Odds Ratio, *CI *Confidence Interval, *CCI *Charlson Comorbidity Index

With regards to the ability to obtain care, there was a numeric difference between private insurance and public-only insurance psoriasis patients but no statistically significant difference (aOR, 0.93; 95% CI, 0.40-2.13; *p *= 0.853). Compared to private insurance patients, uninsured psoriasis patients were significantly more likely to be unable to obtain care (aOR, 3.42; 95% CI, 1.31-8.92; *p *= 0.013). Uninsured psoriasis patients were also significantly more likely to be unable to obtain care than psoriasis patients with public-only insurance (aOR, 3.70; 95% CI, 1.32-10.38; *p* = 0.014).

## Discussion

This cross-sectional, population-based study is among the initial efforts to characterize the relationship between health insurance status and healthcare-seeking behavior in psoriasis patients. In this study, psoriasis patients with public-only insurance were 2.7 times more likely than those with private insurance to delay seeking or receiving care (95% CI, 1.26–5.87). This finding is consistent with trends observed in population-based studies of melanoma patients, which have shown a greater likelihood of delayed surgical treatment in patients with public health insurance than those who are privately insured [20, 30, 31]. There are several possible reasons for our result in psoriasis patients.

Decreased reimbursement rates for physicians seeing publicly insured patients may explain our findings [19, 32–34]. Medicare and Medicaid are the two primary public health insurance programs in the United States. Medicare covers Americans aged 65 and older and some individuals younger than 65 with specific disabilities or conditions, including permanent kidney failure; Medicaid provides coverage for some low-income individuals with limited resources, children, and pregnant women [35]. Prior research has demonstrated a decline in dermatologists’ financial compensation for the evaluation and treatment of Medicare patients [36–38]. One study used current procedural terminology code payments for common dermatology services to assess Medicare reimbursement rates over 20 years [36]. After adjusting for inflation, researchers found that the average dermatologist reimbursement decreased by 10% for non-facility-based services and 18% for facility-based services from 2000 to 2020 [36]. As such, fiscal constraints may limit and subsequently delay access to dermatologic care for Medicare beneficiaries who decide to seek care.

Decreased reimbursement may also influence dermatologists to limit their participation in Medicaid programs, leading to lower acceptance rates of new Medicaid patients than privately insured patients [19, 35, 39–43]. Medicaid programs are coordinated through collaboration between Federal and State governments; therefore, variability exists between states in reimbursement rates and the percentage of dermatologists who accept new Medicaid patients [39, 40, 44, 45]. These differences decrease access to dermatologic care for Medicaid patients compared to privately insured ones, thereby increasing the likelihood of delays in seeking or receiving care.

In addition to reduced reimbursement rates, prolonged appointment wait times may explain our findings. The passage of the Patient Protection and Affordable Care Act in 2010 increased insurance coverage for millions of Americans; however, publicly insured patients may still struggle to access specialist care as quickly as privately insured patients [32–34]. Many Medicaid and Medicare programs rely on forms of managed care to insure their beneficiaries, such as Health Maintenance Organizations (HMOs) [46, 47]. These plans require a primary care physician referral for evaluation by specialists contracted within a specific coverage network [35, 48]. In contrast, non-HMO plans may circumvent this referral process, allowing patients to directly schedule dermatologist appointments irrespective of their coverage network [35]. As a result, patients forced to obtain primary care referrals often interface with the healthcare system multiple times before seeing a dermatologist, leading to potential delays in seeking or receiving care.

Furthermore, wait times tend to differ between dermatologists practicing in academic and private practice settings [19]. Academic dermatologists are more likely to accept publicly insured patients than those in private practice, which potentially increases the number of patients under their care [19]. A greater patient load can prolong appointment wait times for publicly insured patients seeking care in academic settings. Collectively, these factors can delay timely dermatologic care for publicly insured patients compared to privately insured patients [20, 32, 39]. The management of psoriasis requires consistent care, and barriers to access may exacerbate the disease burden and worsen patient outcomes [7, 8, 10, 49].

This study also found that uninsured psoriasis patients were 3.4 times more likely to be unable to obtain care than privately insured patients (95% CI, 1.31–8.92). Compared to psoriasis patients with public-only insurance, uninsured psoriasis patients were also 3.7 times more likely to be unable to obtain care (95% CI, 1.32–10.38). This study did not find significant differences between privately and publicly insured psoriasis patients regarding their ability to obtain care, suggesting that the presence of health insurance is a critical factor to consider when assessing the likelihood of a patient being able to get care. There are several potential explanations for these expected results.

Uninsured patients are less likely to have a regular source of care than those who are insured, as the absence of health insurance limits access to medical services and providers [50, 51]. Since fewer uninsured patients can access care consistently, specifically primary care, many cannot obtain dermatological care, which often requires a referral from a primary care physician [35]. Additionally, since uninsured patients cannot benefit from the cost-sharing conferred by managed care insurance plans, they are more likely to be charged in full and have greater out-of-pocket expenses for their care [50]. When this cost is coupled with the high prices associated with psoriasis care, patients may decide not to seek care for their psoriasis due to the significant economic burden [5, 50, 52].

This study’s findings should be interpreted within the context of the survey design. Data collection in MEPS is self-reported, so responses may be subject to recall bias and disease misclassification [20, 52, 53]. Additionally, MEPS does not assess disease severity in its survey questionnaires [52]. We could not account for the severity of psoriasis in our study population, which may influence healthcare-seeking behavior [5, 52].

## Conclusions

This study found psoriasis patients with public-only insurance were significantly more likely to delay seeking or receiving care than those with private insurance. This study also found that uninsured psoriasis patients were significantly more likely to be unable to obtain care than psoriasis patients with private insurance and those with public-only insurance. Future research should continue to evaluate the influence of health insurance status on healthcare-seeking behavior in psoriasis patients with varying levels of disease severity. Understanding this impact can guide healthcare policy reform that effectively meets the needs of at-risk psoriasis patients and improves patient outcomes. Developing strategies to increase healthcare access is necessary to ensure equitable, timely, and appropriate care for all psoriasis patients, regardless of their insurance status.

## Data Availability

The data utilized by our study is publicly available and de-identified from the Medical Expenditure Panel Survey (MEPS) database between the years 2002-2021, from the following location (doi: https://meps.ahrq.gov/mepsweb/data_stats/download_data_files.jsp). The Public Use File (PUF) numbers for the MEPS Full-Year Consolidated Files are HC-070, HC-079, HC-089, HC-097, HC-105, HC-113, HC-121, HC-129, HC-138, HC-147, HC-155, HC-163, HC-171, HC-181, HC-192, HC-201, HC-209, HC-216, HC-224, and HC-233. The PUF numbers for the MEPS Medical Conditions Files are HC-069, HC-078, HC-087, HC-096, HC-104, HC-112, HC-120, HC-128, HC-137, HC-146, HC-154, HC-162, HC-170, HC-180, HC-190, HC-199, HC-207, HC-214, HC-222, and HC-231.
